# Bulk atmospheric deposition of persistent organic pollutants and polycyclic aromatic hydrocarbons in Central Europe

**DOI:** 10.1007/s11356-019-05464-9

**Published:** 2019-06-14

**Authors:** Barbora Nežiková, Céline Degrendele, Pavel Čupr, Philipp Hohenblum, Wolfgang Moche, Roman Prokeš, Lenka Vaňková, Petr Kukučka, Jakub Martiník, Ondřej Audy, Petra Přibylová, Ivan Holoubek, Peter Weiss, Jana Klánová, Gerhard Lammel

**Affiliations:** 1 Research Centre for Toxic Compounds in the Environment, Masaryk University, Brno, Czech Republic; 2 Umweltbundesamt, Wien, Austria; 3 Multiphase Chemistry Department, Max Planck Institute for Chemistry, Mainz, Germany

**Keywords:** Bulk atmospheric deposition, POPs, PCBs, OCPs, PAHs, Central Europe, Deposition fluxes

## Abstract

Polycyclic aromatic hydrocarbons (PAHs), polychlorinated biphenyls (PCBs) and organochlorine pesticides (OCPs) are ubiquitous and toxic contaminants. Their atmospheric deposition fluxes on the regional scale were quantified based on simultaneous sampling during 1 to 5 years at 1 to 6 background/rural sites in the Czech Republic and Austria. The samples were extracted and analysed by means of gas chromatography coupled to mass spectrometry. For all seasons and sites, total deposition fluxes for Σ_15_PAHs ranged 23–1100 ng m^−2^ d^−1^, while those for Σ6PCBs and Σ12OCPs ranged 64–4400 and 410–7800 pg m^−2^ d^−1^, respectively. Fluoranthene and pyrene were the main contributors to the PAH deposition fluxes, accounting on average for 19% each, while deposition fluxes of PCBs and OCPs were dominated by PCB153 (26%) and *γ*-hexachlorobenzene (30%), respectively. The highest deposition flux of Σ_15_PAHs was generally found in spring, while no seasonality was found for PCB deposition. For deposition fluxes for Σ_12_OCPs, no clear spatial trend was found, confirming the perception of long-lived regional pollutants. Although most OCPs and PCBs hardly partition to the particulate phase in ambient air, on average, 42% of their deposition fluxes were found on filters, confirming the perception that particle deposition is more efficient than dry gaseous deposition. Due to methodological constraints, fluxes derived from bulk deposition samplers should be understood as lower estimates, in particular with regard to those substances which in ambient aerosols mostly partition to the particulate phase.

**Electronic supplementary material:**

The online version of this article (10.1007/s11356-019-05464-9) contains supplementary material, which is available to authorized users.

## Introduction

Polycyclic aromatic hydrocarbons (PAHs) and many halogenated substances, such as polychlorinated biphenyls (PCBs) and organochlorine pesticides (OCPs), are ubiquitous contaminants in the global environment (UNEP 2003). PCBs and OCPs are bioaccumulative and persistent in environmental compartments. Moreover, PCBs, many OCPs and many PAHs are known for their toxic properties, as some compounds in these groups are possibly carcinogenic, mutagenic and teratogenic (Ali et al. 2014; Bansal and Kim 2015; Ludewig and Robertson 2013; Ross 2004). For example, benzo[a]pyrene (BAP) has been classified as carcinogen for humans (group 1), while other PAHs were classified as probable or possible carcinogens (group 2A or 2B) by the International Agency for Research on Cancer (IARC 2010) and are ecotoxic. Most of these substances are now regulated under the auspices of international conventions for the protection of the environment and human health (UNEP 2008; UNECE 1999).

PAHs are products of incomplete combustion and have both anthropogenic (e.g. traffic, domestic heating) and natural sources (e.g. crude oil and wild fires) (Dat and Chang 2017). PCBs were widely used as dielectric fluids, plasticisers and adhesives from 1930s to 1970s (Backe et al. 2002). In Czechoslovakia, the production ended in the year 1984 (Christan and Janse 2005). OCPs were used in agriculture from the middle of the last century and dichlorodiphenyltrichloroethane (DDT) as vector control combatting malaria in tropical countries (el Shahawi et al. 2010).

Persistent organic pollutants (POPs) and PAHs can be found in every compartment of the environment and their physical and chemical characteristics allow them to be transported from one compartment to another (Cetin et al. 2017; Karacik et al. 2013). As semi-volatile organic compounds (SOCs), they are partitioning in the atmosphere between the gaseous and particulate phases. This partitioning is one of the most important factors influencing the fate in the atmosphere of these SOCs (Bidleman 1988; Keyte et al. 2013), and therefore their long-range transport potential.

Exposure of ecosystems to pollutants is dominated by atmospheric depositions, dry and wet. Dry deposition is driven by gravity force and diffusion (Bidleman 1988), while wet deposition is controlled by precipitation rate and intensity (Atlas and Giam 1988; Castro-Jiménez et al. 2015; Staelens et al. 2005). It has been shown that snow is more efficiently scavenging than rain (Lei and Wania 2004; Wania et al. 1998). The effect of forests on deposition into ecosystems was studied, indicating the strong influences of the surface roughness, climate parameters (e.g. wind velocity) and physicochemical properties of the substance (e.g. the octanol/air partition coefficient, *K*_oa_) (McLachlan and Horstmann 1998; Nizzetto et al. 2006; Foan et al. 2012).

Efforts have been made over the last decades to quantify deposition fluxes for various SOCs. Fluxes for large areas or entire regions have been estimated based on multicompartmental modelling (Lammel and Stemmler 2012; Scheringer et al. 2004; Stemmler and Lammel 2012) and based on freshwater sediment pollution (Meijer et al. 2006). It is important to verify the modelling results by direct measurements of atmospheric deposition. There are some studies carried out in quantifying bulk atmospheric deposition of PAHs, PCBs and OCPs experimentally in Europe (e.g. Arellano et al. 2015; Brorström-Lundén et al. 1994; Carrera et al. 2002; Jakobi et al. 2015), while additional data are available for other regions, for example the USA (Schifman and Boving 2015), China (Feng et al. 2017) and the global oceans (González-Gaya et al. 2016). Moreover, additional data for wet deposition and washout ratios are available for Europe (Shahpoury et al. 2015; Škrdlíková et al. 2011).

In previous studies, the seasonal variations of bulk deposition fluxes of PAHs have been characterised, generally showing higher deposition fluxes in winter (Binici et al. 2014; Birgül et al. 2011; Blanchard et al. 2007; Gocht et al. 2007). Seasonal variations of PCBs have also been studied but no clear trend has been observed (Agrell et al. 2002; Blanchard et al. 2007; Brorström-Lundén et al. 1994; Carrera et al. 2002; Newton et al. 2014; Teil et al. 2004). Concerning OCPs, only few studies exist, mainly on the isomers of hexachlorocyclohexane (HCH) (Brorström-Lundén et al. 1994; Carrera et al. 2002; Jakobi et al. 2015; Teil et al. 2004), while the seasonality of OCP atmospheric deposition has not been addressed yet in these studies.

The aim of this study is to provide novel data on atmospheric deposition of PAHs, PCBs and OCPs in Central Europe. In particular, the seasonal and spatial variations of these deposition fluxes were investigated at different rural/background sites along the Czech-Austrian border in 2011–2015.

## Material and methods

### Sampling

Total (wet and dry i.e. bulk) deposition samples were simultaneously collected near the Czech–Austrian border at three sites in the Czech Republic, Kuchařovice (KUC), Košetice (KOS) and Churáňov (CHU), and at three sites in Austria, Wolkersdorf (WOL), Unterbergern (UNT) and Grünbach (GRU). All sites are considered as background with limited anthropogenic sources with the exception of KUC. KUC is a rural site, located in the vicinity of agricultural fields and affected by emissions from residential area (e.g. domestic heating) located near the sampling site (around 100 m). A map of the sampling sites is provided in Figure S1 in the Supplementary Material.

From September 2011 to August 2012, deposition samplers were deployed at each site. Additional deposition samples were also collected at the same three Czech sites during 2012–2015. Exact sampling periods are provided in the Table S1.

The deposition sampler used (Čupr and Pěnkava 2012) consists of a collection vessel (250 mm diameter) made of borosilicate glass, with a stainless steel particulate filter holder located at the bottom of the collection vessel. A glass column containing XAD-2 sorbent (Supelco, USA) is connected to the base of the filter holder and stored in a housing with a moderate heater (Figure S2). The sampler is based on the sampler developed for and successfully applied in the MONARPOP project (Offenthaler et al. 2009; Jakobi et al. 2015). Both samplers are a modification of an earlier design (DIN 2002), improving sampling efficiency and making sure that exclusively inert materials are in contact with the sample. This new design is patented (Čupr and Pěnkava 2012). The XAD resin was pre-cleaned in Soxhlet extractor for 8 h in acetone and 8 h in dichloromethane (DCM), dried overnight and stored at room temperature. The moderate heater was used only when the ambient temperature was lower than 4 °C to prevent the formation of ice and to ensure that snowfall is melted immediately, such that SOC deposition with snowfall is collected and not lost. Therefore, this sampler allows for the simultaneous collection of dry deposited particulate matter and gaseous compounds as well as the wet deposition. Atmospheric particles were collected on a glass microfibre filter (GFF, 70 mm, Whatman, USA) and the dissolved phase was collected on XAD. The sampling duration was about 3 months at each site. From 2013 onwards, GFFs were changed every month. One to three months is the common range of total deposition sampling of POPs (Bergknut et al. 2011; Gocht et al. 2007; Jakobi et al. 2015; Newton et al. 2014). After sampling, all samples were wrapped in aluminium foil and plastic zip lock bags and stored at − 18° C until analysis.

Given the low deposition rates and analytical challenges of SOCs, a sampling period of 3 months is appropriate (e.g. Jakobi et al. 2015). Possible sampling artefacts are photodegradation (photolysis, ozonation), blowoff of deposited particles from the surface (McLachlan 1998) and volatilisation from sampler surfaces (funnel, GFF). The fraction of pollutants dissolved by rainwater (or snow melt) is not subject to volatilisation, but irreversibly trapped in XAD. It had been evaluated using a similar sampler design (funnel, resin cartridge downstream) that up to 10% of the organics contained in the rainwater is not retained by the sampler, but subject to breakthrough (McLachlan and Horstmann 1998) and that up to 10% of polychlorinated dibenzo-*p*-dioxins and -furans present on the surface of the sampler can be lost if the surface is not rinsed after sampling (Horstmann and McLachlan 1997). Similarly, a previous study (funnel, filter and resin cartridge downstream; Franz et al. 1991) has shown that 25–28% of PAHs and 26–62% of PCBs remained on the different parts of the sampler after sampling (i.e. surface, deposition walls) and could be obtained by rinsing the surface. No rinsing was done in this study. Furthermore, because of the significance of surface roughness (McLachlan and Horstmann 1998; Pryor et al. 2007; Glüge et al. 2015), dry particle deposition to artificial surfaces is less efficient than to natural surfaces. Consequently, such type of samplers may underestimate the total flux of the target compounds to terrestrial ecosystems. Nevertheless, various advantages of such type of deposition sampling should be emphasised: comparable sampling across sites and seasons and, particularly, including SOC deposition related to snowfall, and rather low maintenance of device.

### Sample preparation and analysis

GFFs and XAD samples were extracted with DCM using an automatic extractor (Büchi Extraction System, B-811, Switzerland). Surrogate recovery standards (i.e. D8-naphthalene, D10-phenanthrene, D12-perylene, Supelco, Merck, Germany; PCB30 and PCB185, Ultra Scientific, USA) were spiked onto each GFF and XAD before extraction. The extracts were then concentrated using a gentle nitrogen stream. In 2011–2012, one sampler was dedicated to PAH analysis while the second one was for PCBs and OCPs. For the remaining years, PAHs, PCBs and OCPs were analysed from a single deposition sampler and the extracts were divided with 10% used for PAHs and the remaining for PCBs and OCPs. PAHs extracts were transferred to a silica column consisting of 1 g of anhydrous sodium sulphate and 5 g of activated silica and were eluted with 10 mL of *n*-hexane and 20 mL of DCM. Both fractions were collected in the same vial. PCBs and OCPs extracts were transferred to a glass column consisting of 1 g of anhydrous sodium sulphate, 0.5 g of activated silica, 8 g of H_2_SO_4_-modified activated silica and 1 g of activated silica and were eluted with 30 mL of DCM:*n*-hexane (1:1).

The PAHs were analysed using gas chromatography 6890 GC (Agilent Technologies, USA) equipped with a 60 m × 0.25 mm × 0.25 μm DB5-MS column (Agilent Technologies, USA) coupled to a mass spectrometer (MS 5975, Agilent, USA). The temperature programme was 80 °C (1 min), 15 °C min^−1^ to 180 °C, 5 °C min^−1^ to 310 °C (20 min). The inlet temperature was 280 °C. The carrier gas was He with a flow rate of 1.5 mL min^−1^. The temperature of the transfer line was 310 °C and 320 °C for the ion source. The used regime was selected ion monitoring. PCBs and OCPs were analysed using a 7890 GC (Agilent Technologies, USA) coupled to Waters Quattro Micro GC (Waters, USA) equipped with SGE Analytical Science HT-8 (8% Ph) column (60 m × 0.5 mm × 0.25 μm, SGE Analytical Science, Australia) coupled with MS/MS (Agilent Technologies, USA). The temperature programme was 80 °C, 40 °C min^−1^ to 200 °C, 5 °C min^−1^ to 305 °C. The inlet temperature was 280 °C. The carrier gas was helium with a flow rate of 1.5 mL min^−1^. The temperature of the transfer line was 310 °C and of the ion source was 250 °C. The used regime was multiple reaction monitoring. The target compounds in this study were 15 PAHs, acenaphthylene (ACY), acenaphthene (ACE), fluorene (FLN), phenanthrene (PHE), anthracene (ANT), fluoranthene (FLT), pyrene (PYR), benzo(a)anthracene (BAA), chrysene (CHR), benzo(b)fluoranthene (BBF), benzo(k)fluoranthene (BKF), BAP, indeno(1,2,3-cd)pyrene (IPY), dibenz(a,h)anthracene (DHA) and benzo(ghi)perylene (BPE); 6 PCBs, PCB28, PCB52, PCB101, PCB153, PCB138 and PCB180; and 12 OCPs, namely 4 HCH isomers (*α, β*, *γ*, *δ*), 6 DDX compounds i.e. *o*,*p'*- and p,p’-DDT, dichlorodiphenyldichloroethene (DDE) and dichlorodiphenyldichloroethane (DDD) and penta- and hexachlorobenzene (PeCB and HCB).

### QA-QC

No field blanks were collected within this study, but two field blanks, each consisting of XAD and GFF, from a following, methodologically identical study, were used instead. These blank levels of individual PAHs, PCBs and OCPs were below the limit of detection or low otherwise, suggesting minor contamination during sampling, transport and analysis. Mean blank values with standard deviations are provided in Table S2. There were also five solvent blanks analysed for PAHs and three solvent blanks for PCBs and OCPs, which showed levels below the detection limit except for PHE, FLT, PYR, *α*-HCH and *γ*-HCH, which had levels lower than the instrumental limit of quantification (iLOQ). iLOQs were defined from the instruments as a signal to noise ratio of ten for the lowest point of the calibration curve and are presented in Table S3.

The recoveries of individual samples were ranging from 55.6 to 117.2% for PAHs and from 63.1 to 109.1% for PCBs and OCPs. The reported fluxes have not been adjusted for recoveries but were blank corrected, by subtracting the mean concentrations of SOCs in the field blanks. To this end, blank values < iLOQ were replaced by 0. For derivation of temporal averages, values were replaced by iLOQ/2 whenever the determined concentrations in samples were lower than iLOQ.

## Results and discussion

### Atmospheric bulk deposition flux of PAHs, PCBs and OCPs and their composition profiles

The total deposition fluxes for 15 PAHs (Σ_15_PAHs) total deposition fluxes ranged at 23 to 1100 ng m^−2^ d^−1^ with an average value of 190 pg m^−2^ d^−1^ for all seasons and sites investigated (Table S4). The here found PAH deposition fluxes are comparable with previous studies from remote or rural sites (i.e. 38–2000 ng m^−2^ d^−1^, see Table 1), but generally lower than those reported from urban sites (i.e. 36–20000 ng m^−2^ d^−1^, see Table 1). This is due to the stronger influence of PAH primary sources (e.g. road traffic, fossil fuel burners for heating) in urban areas compared to remote or rural areas. PAHs have been effectively mitigated across Europe in recent decades (EEA 2017). However, direct comparisons with other studies should be done with caution, given the different PAHs considered (in this study, *N* = 15 and in others *N* = 7–23).

**Table 1:** Overview of bulk deposition fluxes of PAHs, OCPs and PCBs reported from Europe and the Mediterranean. ^a^Different number of congeners were measured. ^b^Only p,p’ isomers were measured

Site, year of sampling	Σ_7_PCB (pg m^−2^ d^−1^)	HCB (pg m^−2^ d^−1^)	Σ_4_HCH (ng m^−2^ d^−1^)	Σ_6_DDX (pg m^−2^ d^−1^)	Σ_*n*_PAH (ng m^−2^ d^−1^)	Type of sampler	Reference
a. Remote							
Sweden; 1996–2005	190–2300		0.7–11^(*α* + *γ*)^	40–670^b^	20–390 ^(*n* = 12)^	Teflon-coated surface	Hansson et al. 2006
Andorra, Norway, Switzerland; 1997–1998					38–63 ^(*n* = 23)^	Stainless steel bucket	Fernández et al. 2003
Andorra, Norway, Switzerland; 1997–1998	1033–33,00	47–500	5–16			Stainless steel bucket	Carrera et al. 2002
Italy; 2003	553–18,217					Glass bottle	Nizzetto et al. 2006
Austria, Spain, Scotland, Slovakia; 2004–2006	3733–16,267	33–400	0.6–2 ^(*α* + *γ*)^			Teflon or stainless steel reservoir	Arellano et al. 2015
Austria, Germany, Switzerland; 2005–10		110–211	2–6 ^(*α* + *γ*)^	395–1586		Isolated funnel-adsorber system	Jakobi et al. 2015
Sweden; 2006–2009	570–10,000					Isolated funnel-adsorber system	Bergknut et al. 2011
Sweden; 2009–2010	2700–6333		0.5–4 ^(*α* + *γ*)^			Isolated funnel-adsorber system	Newton et al. 2014
Austria, Czech Republic, 2011–2015	73–2030^a^	29–270	0.1–4	48–750	23–660 ^(*n* = 15)^	Isolated funnel-adsorber system	*This study*
b. Rural							
Sweden; 1989–1990	2300–4500	70–2700	1–40 ^(*α* + *β* + *γ*)^		210–2000 ^(*n* = 11)^	Glass collector	Brorström-Lundén et al. 1994
Baltic sea; 1990–1993	270–171,000					PUF plugs	Agrell et al. 2002
Germany; 2001–2002					334–1063 ^(*n* = 17)^	Isolated funnel-adsorber system	Gocht et al. 2007
Czech Republic, 2011–2013	64–4400^a^	47–230	0.3–2	490–5030	49–1100 ^(*n* = 15)^	Isolated funnel-adsorber system	*This study*
c. Urban							
United Kingdom; 1992–1992					1310–18,200 ^(*n* = 13)^	Glass vessel	Halsall et al. 1997
France; 1999–2000					177–2100 ^(*n* = 14)^	Aluminium bottle	Ollivon et al. 2002
France; 1999–2003	7671–12,0547				36–622 ^(*n* = 14)^	Aluminium bottle	Blanchard et al. 2007
Italy; 2002–2004					221–10,575 ^(*n* = 17)^	Pyrex bottle	Rossini et al. 2007
Turkey; 2005					260–19,500 ^(*n* = 15)^	Stainless steel pot	Esen et al. 2008
Italy; 2005					101–693 ^(*n* = 15)^	Stainless steel vessel	Gambaro et al. 2009
Turkey; 2006–2007			0.2–15 ^(*β* + *γ*)^		11,600 ^(*n* = 10)^	Dry and wet collector	Binici et al. 2014
Turkey; 2008–2009			63			Stainless steel pot	Cindoruk and Tasdemir 2014
Italy; 2007–2008					92–2432 ^(*n* = 7)^	Glass bottle	Amodio et al. 2014
Turkey; 2008–2009					11,400 ^(*n* = 12)^	Dry and wet collector	Birgül et al. 2011
Italy; 2015–2016	75–1220^a^	200–650			20–280 ^(*n* = 20)^	Isolated funnel-adsorber system	Qu et al. 2019

**Fig. 1:**
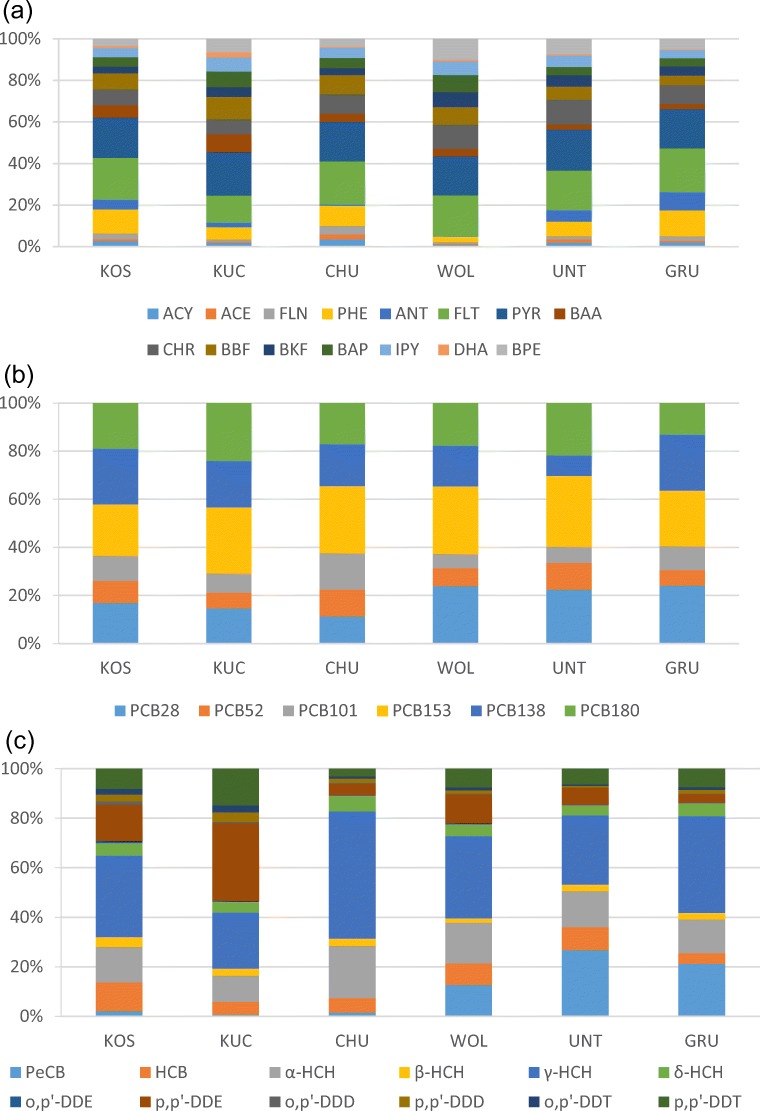
**a** PAH, **b** PCB and **c** OCP patterns across sites

In our study, FLT and PYR were generally the main contributors to PAHs’ deposition fluxes, accounting on average for 19% both (Fig. 1). There is one exception at KUC where in some samples (from autumn 2012 to summer 2013) BBF is also a significant contributor accounting on average for 17% of the total flux of PAHs. The possible reason is because of the different nature of this site, i.e. rural while all others are background, which may reflect differences in emissions. Many other studies also reported FLT and PYR as the main contributors of ΣPAHs, with non-negligible contributions from PHE and CHR (Birgül et al. 2011; Blanchard et al. 2007; Esen et al. 2008; Fernández et al. 2003; Gambaro et al. 2009; Gocht et al. 2007; Halsall et al. 1997; Ollivon et al. 2002; Rossini et al. 2007).

For all seasons and sites investigated, total deposition fluxes for 6 PCBs (Σ_6_PCBs) and 12 OCPs (Σ_12_OCPs) measured were 64–4400 (average value of 400 pg m^−2^ d^−1^) and 410–7800 pg m^−2^ d^−1^ (average 1900 pg m^−2^ d^−1^), respectively (Table S4). These results are within the ranges spanned by other studies in Europe (Table 1). Bulk deposition of HCH was reported up to one order of magnitude higher from Switzerland in the 1990s (Table 1), in accordance with emission reductions achieved (EEA 2017). The results suggest that atmospheric deposition in the 2010s is an important pathway of pollution transfer to ecosystems in the Central European background.

Wet deposition was expected to be most relevant for substances partitioning to the particulate phase or gaseous, but with significant water solubility. At KOS, a significant correlation (*p* < 0.05) between the total deposition mass flux and precipitation amount is found only for the two HCH isomers. This test was applied only for the KOS data subset, because of its size (*N* = 17, while *N* = 4 or *N* = 8 for the other sites; *N* is the number of samples). The result supports the perception of influence of water solubility/air-water equilibrium (see also below, Henry coefficients of *α*- and *γ*-HCH are 0.7 and 0.3 Pa m^3^ mol^−1^, respectively).

The deposition mass fluxes of PCBs were dominated by PCB153 (26%, Fig. 1). Next main contributors were PCB28, PCB138 and PCB180, depending on locations. This is in agreement with Agrell et al. (2002), but in contradiction with other studies in Europe which reported that PCBs deposition fluxes were dominated by lower molecular weight PCBs, specifically PCB28, PCB52 and PCB101 (Bergknut et al. 2011; Carrera et al. 2002; Newton et al. 2014; Teil et al. 2004). Such spatial variation of the substance pattern upon deposition is expected as resulting from the spatial variability of precipitation and temperature, the latter influencing all intercompartmental processes (Stemmler and Lammel 2012; Wania et al. 2003; Wania and Westgate 2008).

The deposition fluxes of OCPs were generally dominated by γ-HCH, accounting on average for 30% of Σ_12_OCPs, at CHU for even 52%. Second to most contributed α-HCH to the deposition flux of OCPs, accounting on average for 15%. From winter 2011/12 until winter 2013/14, HCH concentration in air of KOS were 46% of the concentration of the DDT compounds (Shahpoury et al. 2015), but the ratio of total deposition fluxes of these pollutants during this period was *F*_HCH_/*F*_DDX_ = 2.4. One decade earlier, HCH abundance in air at KOS was measured ≈ 50% higher than the one of DDX, but more than one order of magnitude higher in rainwater collected in KOS (Holoubek et al. 2007). This comparison clearly points to a much more effective wet scavenging of HCH vs. DDT compounds, explained by the lower Henry coefficient − 0.7 and 0.3 Pa m^3^ mol^−1^ for *α*- and *γ*-HCH, respectively, vs. 33 and 1.1 Pa m^3^ mol^−1^ for *p,p’*-DDE and -DDT, respectively (298 K; Jantunen and Bidleman 2006; Shen and Wania 2005; Xiao et al. 2004). Together with the correlation with precipitation amounts in KOS (above), it also supports the perception that the total deposition flux of OCPs is dominated by wet deposition. A prevalence of γ-HCH and α-HCH among deposited OCPs had already been reported and attributed to abundance in air, but also wet scavenging (Carrera et al. 2002; Newton et al. 2014; Cindoruk and Tasdemir 2014; Jakobi et al. 2015). These results were consistent among the different sites, except for KUC where the flux of OCPs was dominated by *p,p'*-DDE accounting on average for 31%. This may reflect the different land use of the past, given that DDT was used for agricultural purposes in this region of the Czech Republic until the 1970s.

### Seasonal and spatial variations

**Fig. 2:**
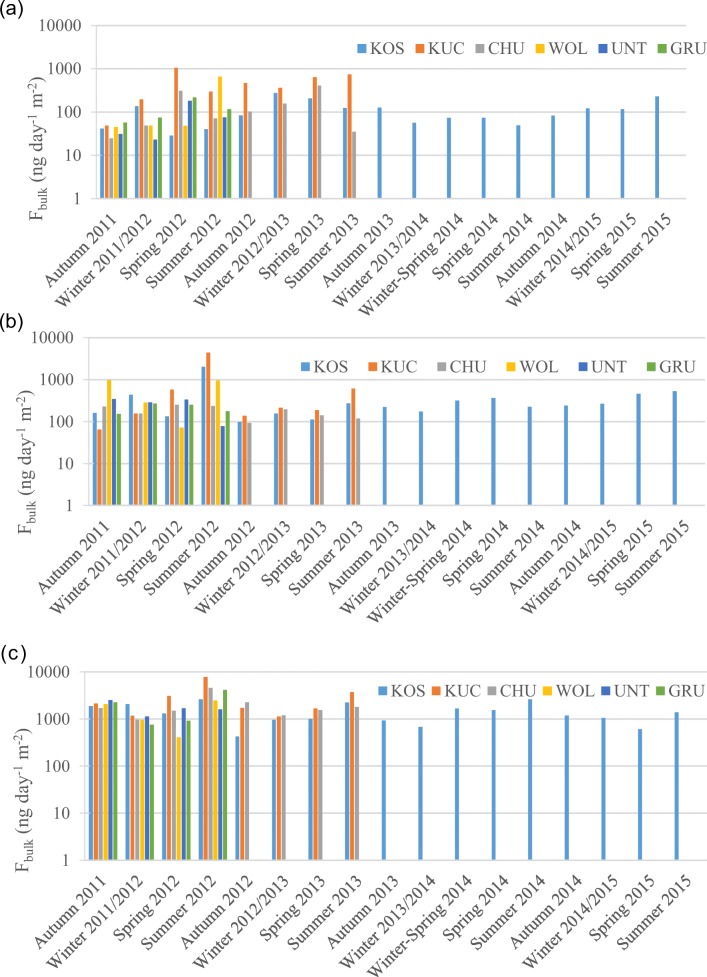
Bulk deposition fluxes of **a** 15 PAHs, **b** 6 PCBs and **c** 12 OCPs shown in log axis

Significantly (*p* < 0.05) higher PAH deposition fluxes were observed at KUC than at the other sites. The spatial variation across the other sites, apart from KUC, was lower for PAHs than for PCBs. The maximum at the rural site KUC was expected, because of local emissions, namely domestic heating from a nearby village with road traffic and agricultural machinery, unlike at the other sites. The PAH deposition flux at KUC site ranged from 49 to 1100 ng m^−2^ d^−1^ with an average of 140 ng m^−2^ d^−1^, i.e. 2–6 times higher than for the other sites (Fig. 2, Table S4). The highest flux of PAHs at KUC was observed in spring-summer, while at KOS, it was highest in autumn–winter and at WOL it was highest in summer. These results are somewhat unexpected, because the concentration of PAHs in the air are higher in winter everywhere (Dat and Chang 2017), which results from the combination of several factors i.e. increased sources (i.e. domestic heating), meteorological conditions (i.e. lower height of mixed layer; Finlayson-Pitts and Pitts 2000; Dvorská et al. 2012) and longer photochemical lifetime (Finlayson-Pitts and Pitts 2000; Dvorská et al. 2012). Obviously, the seasonal variations of PAH bulk deposition is not dominated by atmospheric concentration throughout the region, but influenced by other, spatially variable factors, such as meteorological. Also, earlier studies (Dickhut and Gustafson 1995; Fernández et al. 2003) explained the seasonality of the bulk deposition flux by the seasonality of precipitation. In the here studied region, highest precipitation was generally observed in summer (Table S1). Most of the studies (Halsall et al. 1997; Ollivon et al. 2002; Blanchard et al. 2007; Gocht et al. 2007; Esen et al. 2008; Birgül et al. 2011; Binici et al. 2014) reported highest levels of PAHs flux in winter. Moreover, we note that the influence of open fires on the PAH time series cannot be excluded.

The PAH bulk deposition time series in KOS, ≈4 years, does not show a downward trend (Table S4, Fig. 2). A downward trend could reflect and would eventually confirm ongoing mitigation measures (EEA 2017). Because of interannual variation of precipitation and other environmental parameters, much longer time series would be needed to identify a significant long-term trend.

Significantly (*p* < 0.05) higher deposition flux of Σ_6_PCB was found at KUC, where it ranged from 64 to 4400 pg m^−2^ d^−1^ with an average of 140 pg m^−2^ d^−1^, i.e. which is 1.4–4.5 times higher than for the other sites (Table S4). This was expected because of the rather intense historical use of PCBs in this region (electrical equipment and constructions; Christan and Janse 2005). Regarding the seasonal variations, a higher flux of Σ_6_PCBs was generally measured in summer in KOS, KUC and WOL, while the lowest mass flux to all sites was generally measured in autumn. At the other sites, no obvious seasonal variation was found. There is a peak in summer 2012 in KOS. The possible reason is the construction of a meteorological tower at the observatory, distanced of about 100 m from the sampling site that may have enhanced revolatilisation from soils. PCB deposition fluxes were reported with varying seasonality from sites across Europe (Brorström-Lundén et al. 1994; Agrell et al. 2002; Carrera et al. 2002; Teil et al. 2004; Nizzetto et al. 2006; Blanchard et al. 2007; Bergknut et al. 2011; Newton et al. 2014).

The total deposition fluxes for Σ_12_OCPs observed for the different sites (Fig. 2, Table S4) were not statistically different from each other. This supports the perception of long-lived (persistent), regionally distributed pollutants. However, there was one exception, namely PeCB was much higher at the Austrian sites (averages ranging 110–250 pg m^−2^ d^−1^) than at the Czech sites (averages ranging 12–29 pg m^−2^ d^−1^, same time period; Fig. 1c). The reason is unknown.

The highest flux of Σ_12_OCPs was generally measured in summer, but without significant seasonal variation. Jakobi et al. (2015) reported higher deposition fluxes in summer for HCB and HCH in Central Europe, but obvious differences across sites for DDX compounds, similar to this study. The variability was attributed to different origin of air masses advected to the sites. Arellano et al. (2015) also reported higher flux of HCH in spring–summer in Slovakia. Newton et al. (2014) did not find an obvious seasonal variation.

The ratio α-HCH/γ-HCH was not significantly different across sites (*p* < 0.05) and smaller than 1 in all seasons at all sites. This suggests using lindane rather than technical HCH. The fraction of 5 ring PAHs among all measured PAHs was not significantly different (*p* < 0.05) across sites with one exception, comparing GRU and WOL. These two ratios suggest that pollutants are distributed equally in Central Europe. The ratio between sum of all DDT isomers over all isomers of DDX compounds was significantly different (*p* < 0.05) between KOS and all other sites with one exception, GRU. This ratio was highest at KOS, suggesting most recent usage of DDT in that area. The ratio between *o*,*p* isomers and all isomers of DDX compounds was significantly different (*p* < 0.05) between KOS and all sites, also, between KUC and CHU, GRU and then between UNT and GRU. These results must be viewed with caution for the Austrian sites, because of the low number (*N* = 4) of samples.

### Distribution between XAD and GFF

The sampler used was designed such that the freely dissolved phase was collected on XAD while the particulate phase was collected on GFF. Therefore, the fraction observed on GFF will be described using the particulate fraction from deposition (*θ*_dep_), which should not be confused with the particulate mass fraction in aerosols (*θ*). It is known that for apolar or mid-polar compounds particle, scavenging is more efficient than gas scavenging (Bidleman 1988); therefore, we can expect that *θ*_dep_ will be higher than *θ*. However, we cannot exclude sampling artefacts affecting the accuracy of *θ*_dep_, as discussed above (“Material and methods”).

**Fig. 3:**
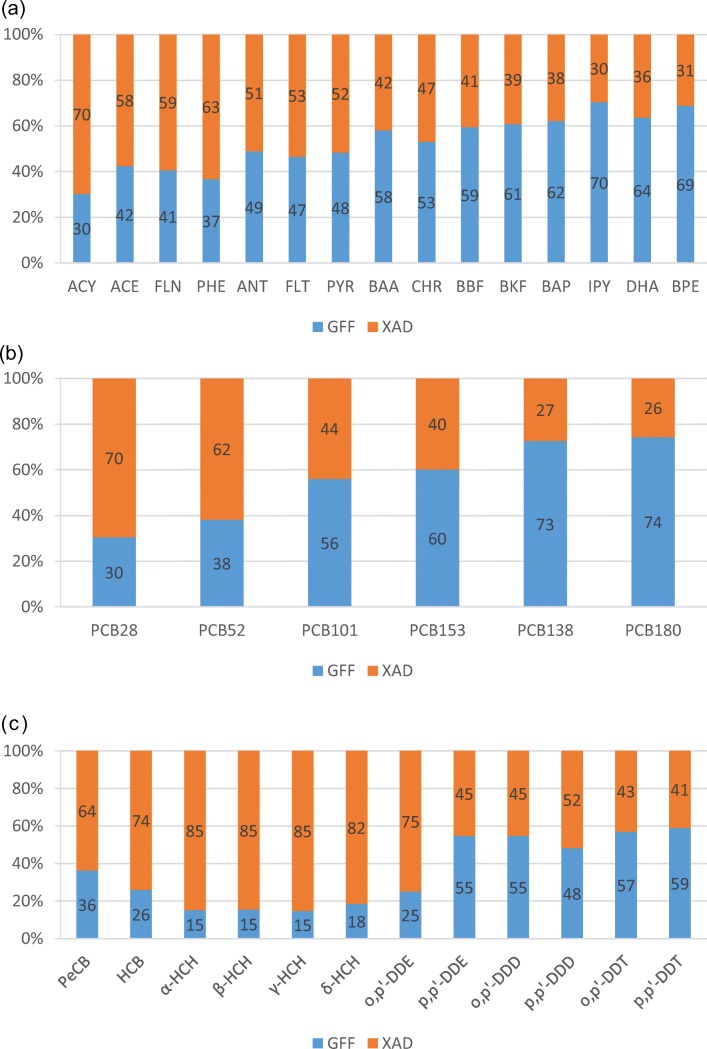
Average distribution between GFF and XAD of **a** PAHs, **b** PCBs and **c** OCPs

Only measurements for which SOCs were > iLOQ in both GFF and XAD are considered. For PAHs and PCBs, *θ*_dep_ generally increased with increasing molecular weight and/or decreasing volatility (Fig. 3). However, this was not observed for OCPs (Fig. 3). Moreover, *θ*_dep_ was generally higher in summer than in winter for all groups of compounds (Figure S3). This implies that the particulate mass fraction in aerosols, *θ*, which shows the opposite seasonality (e.g. for PAHs and OCPs in the study region: Shahpoury et al. 2015; Degrendele et al. 2014), is not preserved in total deposition. The same shift was already reported for wet deposition fluxes from KOS (Škrdlíková et al. 2011). The seasonal variations of cloud depth and cloud base height, and of the scavenging efficiencies of rain and snow for gases and particles (Bidleman 1988; Škrdlíková et al. 2011; Wania and Westgate 2008), as well as of wind velocity, influencing dry particle deposition (Pryor et al. 2007), may well explain the finding.

Apart from environmental parameters, sampling artefacts such as temperature-driven volatilisation from the surface of the sampler, stronger in summer, could have shifted *θ*_dep_ as observed. Also, less precipitation in winter (Table S1) may have caused a negative sampling artefact (i.e. collected particulate material not efficiently washed from the surface of the sampler to the sampling media), eventually contributed to shifted *θ*_dep_ as observed.

## Conclusions

We studied atmospheric bulk deposition of a number of organic pollutants at 6 sites in Central Europe during 2011–2015. The time series (1–≈ 4 years) are too short, to address long-term trends. The results suggest that atmospheric deposition in 2010 is an important pathway of pollution transfer to ecosystems in the Central European background. The substance patterns are quite similar across sites (except for one, the rural site, which is explained by historical usage/pollution of HCH and DDT; and except relatively higher PeCB deposition at the Austrian sites).

For substances which deposition is dominated by dry particle deposition, and because of the significance of surface roughness for dry particle deposition (McLachlan and Horstmann 1998; Pryor et al. 2007; Glüge et al. 2015), the observed patterns may deviate significantly from the substance patterns the natural surfaces (grassland, cropland, forest) are subject to. This also implies a systematic underestimate of the flux of those pollutants which mostly partition to particulate matter in ambient aerosols. For this reason and because of the negative sampling artefact arising from volatilisation losses of dry deposited gaseous substances from the sampler surface, fluxes derived from bulk deposition samplers in use should be understood as lower estimates of the flux into terrestrial ecosystems. This underestimate is significant as dry deposition is more efficient than wet deposition for PAHs (Škrdlíková et al. 2011) and, considering substance properties (*K*_oa_, besides other; Wania and Westgate 2008), even more so for PCBs and OCPs. While the volatilisation losses might be unavoidable for long sampling periods, the dry particle deposition efficiency could be mimicked more realistic by a sampler design with higher surface roughness, e.g. mimicking the surface roughness of particularly forest, but also cropland or grassland. Similarly, deposition to stone façades had been mimicked using a sampler surface with the identical roughness as the façade under study (Lammel and Metzig 1997).

## Electronic supplementary material

ESM 1 (PDF 798 kb)

## References

[CR1] Agrell C, Larsson P, Okla L, Agrell J (2002). PCB congeners in precipitation, wash out ratios and depositional fluxes within the Baltic Sea region, Europe. Atmos Environ.

[CR2] Ali U, Syed JH, Malik RN, Katsoyiannis A, Li J, Zhang G, Jones KC (2014). Organochlorine pesticides (OCPs) in South Asian region: a review. Sci Total Environ.

[CR3] Amodio M, de Gennaro G, di Gilio A, Tutino M (2014). Monitoring of the deposition of PAHs and metals produced by a steel plant in Taranto (Italy). Adv Meteorol.

[CR4] Arellano L, Fernández P, Fonts R, Rose NL, Nickus U, Thies H, Stuchlík E, Camarero L, Catalan J, Grimalt JO (2015). Increasing and decreasing trends of the atmospheric deposition of organochlorine compounds in European remote areas during the last decade. Atmos Chem Phys.

[CR5] Atlas E, Giam CS (1988). Ambient concentration and precipitation scavenging of atmospheric organic pollutants. Water Air Soil Pollut.

[CR6] Backe C, Larsson P, Agrell C (2002). Spatial and temporal variation of polychlorinated biphenyl (PCB) in precipitation in southern Sweden. Sci Total Environ.

[CR7] Bansal V, Kim KH (2015). Review of PAH contamination in food products and their health hazards. Environ Int.

[CR8] Bergknut M, Laudon H, Jansson S, Larsson A, Gocht T, Wiberg K (2011). Atmospheric deposition, retention, and stream export of dioxins and PCBs in a pristine boreal catchment. Environ Pollut.

[CR9] Bidleman TF (1988). Atmospheric processes. Environ Sci Technol.

[CR10] Binici B, Yenisoy-Karakaş S, Bilsel M, Durmaz-Hilmioglu N (2014). Sources of polycyclic hydrocarbons and pesticides in soluble fraction of deposition samples in Kocaeli, Turkey. Environ Sci Pollut Res.

[CR11] Birgül A, Tasdemir Y, Cindoruk SS (2011). Atmospheric wet and dry deposition of polycyclic aromatic hydrocarbons (PAHs) determined using a modified sampler. Atmos Res.

[CR12] Blanchard M, Teil MJ, Guigon E, Larcher-Tiphagne K, Ollivon D, Garban B, Chevreuil M (2007). Persistent toxic substance inputs to the river Seine basin (France) via atmospheric deposition and urban sludge application. Sci Total Environ.

[CR13] Brorström-Lundén E, Lindskog A, Mowrer J (1994). Concentrations and fluxes of organic compounds in the atmosphere of the Swedish west coast. Atmos Environ.

[CR14] Carrera G, Fernández P, Grimalt JO, Ventura M, Camarero L, Catalan J, Nickus U, Thies H, Psenner R (2002). Atmospheric deposition of organochlorine compounds to remote high mountain lakes of Europe. Environ Sci Technol.

[CR15] Castro-Jiménez J, Dachs J, Eisenreich SJ (2015) Atmospheric deposition of POPs: Implications for the chemical pollution of aquatic environments. In: Zeng EY, Barcelo D (eds) Persistent Organic Pollutants (POPs): Analytical Techniques, Environmental Fate and Biological Effects, Chapter 8, vol 67. Elsevier - Comprehensive Analytical Chemistry, pp 295–322. 10.1016/B978-0-444-63299-9.00008-9

[CR16] Cetin B, Öztürk F, Keles M, Yurdakul S (2017). PAHs and PCBs in an Eastern Mediterranean megacity, Istanbul: their spatial and temporal distributions, air-soil exchange and toxicological effects. Environ Pollut.

[CR17] Christan E, Janse J (2005) EuroPCB: inventory PCB enforcement in member states. Part II: Fiches - Results for each member state. Inspectorate of the Ministry of Housing, Spatial Planning and the Environment South Unit of the Netherlands, vol 68, Vienna http://www.cleen-europe.eu/file/download/71/EuroPCB_part_II_fiches_ final.pdf. Accessed 4 Feb 2019

[CR18] Cindoruk SS, Tasdemir Y (2014). The investigation of atmospheric deposition distribution of organochlorine pesticides (OCPs) in Turkey. Atmos Environ.

[CR19] Čupr P, Pěnkava B (2012). Vzorkovač atmosférické depozice (Atmospheric deposition sampler). Patent. No. 23347. (owner: Masarykova univerzita, CZ, BAGHIRRA s.r.o. Praha, CZ).

[CR20] Dat ND, Chang MB (2017). Review on characteristics of PAHs in atmosphere, anthropogenic sources and control technologies. Sci Total Environ.

[CR21] Degrendele C, Okonski K, Melymuk L, Landlová L, Kukučka P, Čupr P, Klánová J (2014). Size specific distribution of the atmospheric particulate PCDD/Fs, dl-PCBs and PAHs on a seasonal scale: implications for cancer risks from inhalation. Atmos Environ.

[CR22] Dickhut RM, Gustafson KE (1995). Atmospheric washout of polycyclic aromatic hydrocarbons in the Southern Chesapeake Bay region. Environ Sci Technol.

[CR23] DIN (2002). German Industrial Standard. Luftbeschaffenheit und Bodenbeschaffenheit - Messen der atmosphärischen Deposition organischer Spurenstoffe; trichter-adsorber-verfahren - Teil 1: Sammelgeräte; Anforderungen, Aufbau, Anwendung. DIN.

[CR24] Dvorská A, Komprdová K, Lammel G, Klánová J, Plachá H (2012). Polycyclic aromatic hydrocarbons in background air in central Europe - seasonal levels and limitations for source apportionment. Atmos Environ.

[CR25] EEA (2017) European Union Emission Inventory Report 1990–2016 under the UNECE Convention on Long-Range Transboundary Air Pollution (LRTAP).

[CR26] el Shahawi MS, Hamza A, Bashammakh AS, al Saggaf WT (2010). An overview on the accumulation, distribution, transformations, toxicity and analytical methods for the monitoring of persistent organic pollutants. Talanta.

[CR27] Esen F, Cindoruk SS, Tasdemir Y (2008). Bulk deposition of polycyclic aromatic hydrocarbons (PAHs) in an industrial site of Turkey. Environ Pollut.

[CR28] Feng D, Liu Y, Gao Y, Zhou J, Zheng L, Qiao G, Ma L, Lin Z, Grathwohl P (2017). Atmospheric bulk deposition of polycyclic aromatic hydrocarbons in Shanghai: temporal and spatial variation, and global comparison. Environ Pollut.

[CR29] Fernández P, Carrera G, Grimalt JO, Ventura M, Camarero L, Catalan J, Nickus U, Thies H, Psenner R (2003). Factors governing the atmospheric deposition of polycyclic aromatic hydrocarbons to remote areas. Environ Sci Technol.

[CR30] Finlayson-Pitts BJ, Pitts JN (2000). Chemistry of the upper and lower atmosphere.

[CR31] Foan L, Domercq M, Bermejo R, Santamaria JM, Simon V (2012). Polycyclic aromatic hydrocarbons (PAHs) in remote bulk and throughfall deposition: seasonal and spatial trends. Environ Eng Manag J.

[CR32] Franz TP, Eisenreich SJ, Swanson MB (1991). Evaluation of precipitation samplers for assessing atmospheric fluxes of trace organic contaminants. Chemosphere.

[CR33] Gambaro A, Radaelli M, Piazza R, Stortini AM, Contini D, Belosi F, Zangrando R, Cescon P (2009). Organic micropollutants in wet and dry depositions in the Venice Lagoon. Chemosphere.

[CR34] Glüge J, Bogdal C, Scheringer M, Hungerbühler K (2015). Atmospheric gas-particle partitioning versus gaseous/particle-bound deposition of SVOCs: why they are not equivalent. Atmos Environ.

[CR35] Gocht T, Klemm O, Grathwohl P (2007). Long-term atmospheric bulk deposition of polycyclic aromatic hydrocarbons (PAHs) in rural areas of Southern Germany. Atmos Environ.

[CR36] González-Gaya B, Fernández-Pinos MC, Morales L, Méjanelle L, Abad E, Piña B, Duarte CM, Jiménez B, Dachs J (2016). High atmosphere-ocean exchange of semivolatile aromatic hydrocarbons. Nat Geosci.

[CR37] Halsall CJ, Coleman PJ, Jones KC (1997). Atmospheric deposition of polychlorinated dibenzo-*p*-dioxins/dibenzofurans (PCDD/Fs) and polycyclic aromatic hydrocarbons (PAHs) in two UK cities. Chemosphere.

[CR38] Hansson K, Palm Cousins A, Brorström-Lundén E (2006). Atmospheric concentrations in air and deposition fluxes of POPs at Råö and Pallas, trends and seasonal and spatial variations.

[CR39] Holoubek I, Klánová J, Jarkovský J, Kohoutek J (2007). Trends in background levels of persistent organic pollutants at Kosetice observatory, Czech Republic. Part I. Ambient air and wet deposition 1996-2005. J Environ Monit.

[CR40] Horstmann M, McLachlan MS (1997). Sampling bulk deposition of polychlorinated dibenzo-p-dioxins and dibenzofurans. Atmos Environ.

[CR41] IARC (2010). Some non-heterocyclic polycyclic aromatic hydrocarbons and some related exposures. IARC Monographs on the Evaluation of Carcinogenic Risks to Humans Vol. 92.

[CR42] Jakobi G, Kirchner M, Henkelmann B, Körner W, Offenthaler I, Moche W, Weiss P, Schaub M, Schramm KW (2015). Atmospheric bulk deposition measurements of organochlorine pesticides at three alpine summits. Atmos Environ.

[CR43] Jantunen LM, Bidleman TF (2006). Henry’s law constants for hexachlorobenzene, p,p′-DDE and components of technical chlordane and estimates of gas exchange for Lake Ontario. Chemosphere.

[CR44] Karacik B, Okay OS, Henkelmann B, Pfister G, Schramm KW (2013). Water concentrations of PAH, PCB and OCP by using semipermeable membrane devices and sediments. Mar Pollut Bull.

[CR45] Keyte IJ, Harrison RM, Lammel G (2013). Chemical reactivity and long-range transport potential of polycyclic aromatic hydrocarbons - a review. Chem Soc Rev.

[CR46] Lammel G, Metzig G (1997). Pollutant fluxes onto the façades of a historical monument. Atmos Environ.

[CR47] Lammel G, Stemmler I (2012). Fractionation and current time trends of PCB congeners: evolvement of distributions 1950-2010 studied using a global atmosphere-ocean general circulation model. Atmos Chem Phys.

[CR48] Lei YD, Wania F (2004). Is rain or snow a more efficient scavenger of organic chemicals?. Atmos Environ.

[CR49] Ludewig G, Robertson LW (2013). Polychlorinated biphenyls (PCBs) as initiating agents in hepatocellular carcinoma. Cancer Lett.

[CR50] McLachlan MS, Lükewille A (1998). Basic consideration and practical experience in the development of methods to measure atmospheric deposition of persistent organic pollutants. EMEP experts meeting on measurements of persistent organic pollutants (POPs) in air and precipitation.

[CR51] McLachlan MS, Horstmann M (1998). Forests as filters of airborne organic pollutants: a model. Environ Sci Technol.

[CR52] Meijer SN, Dachs J, Fernandez P, Camarero L, Catalan J, Del Vento S, van Drooge B, Jurado E, Grimalt JO (2006). Modelling the dynamic air–water–sediment coupled fluxes and occurrence of polychlorinated biphenyls in a high altitude lake. Environ Pollut.

[CR53] Newton S, Bidleman T, Bergknut M, Racine J, Laudon H, Giesler R, Wiberg K (2014). Atmospheric deposition of persistent organic pollutants and chemicals of emerging concern at two sites in northern Sweden. Environ Sci Process Impacts.

[CR54] Nizzetto L, Cassani C, di Guardo A (2006). Deposition of PCBs in mountains: the forest filter effect of different forest ecosystem types. Ecotoxicol Environ Saf.

[CR55] Offenthaler I, Jakobi G, Kaiser A, Kirchner M, Krauchi N, Niedermoser B, Schramm KW, Sedivy I, Staudinger M, Thanner G, Weiss P, Moche W (2009). Novel sampling methods for atmospheric semi-volatile organic compounds (SOCs) in a high altitude alpine environment. Environ Pollut.

[CR56] Ollivon D, Blanchoud H, Motélay-Massei A, Garban B (2002). Atmospheric deposition of PAHs to an urban site, Paris, France. Atmos Environ.

[CR57] Pryor SC, Larsen SE, Sorensen LL, Barthelmie RJ, Grönholm T, Kulmala M, Launiainen S, Rannik U, Vesala T (2007) Particle fluxes over forests: analyses of flux methods and functional dependencies. J Geophys Res 112. 10.1029/2006JD008066

[CR58] Qu C, Albanese S, Lima A, Hope D, Pond P, Fortelli A, Romano N, Cerino P, Pizzolante A, De Vivo B (2019). The occurrence of OCPs, PCBs, and PAHs in the soil, air, and bulk deposition of the Naples metropolitan area, southern Italy: implications for sources and environmental processes. Environ Int.

[CR59] Ross G (2004). The public health implications of polychlorinated biphenyls (PCBs) in the environment. Ecotoxicol Environ Saf.

[CR60] Rossini P, Matteucci G, Raccanelli S, Favotto M, Guerzoni S, Gattolin M (2007). Polycyclic aromatic hydrocarbons in atmospheric depositions around the Venice Lagoon. Polycycl Aromat Compd.

[CR61] Scheringer M, Stroebe M, Wania F, Wegmann F, Hungerbühler K (2004). The effect of export to the deep sea on the long-range transport potential of persistent organic pollutants. Environ Sci Pollut Res.

[CR62] Schifman LA, Boving TB (2015). Spatial and seasonal atmospheric PAH deposition patterns and sources in Rhode Island. Atmos Environ.

[CR63] Shahpoury P, Lammel G, Holubová Šmejkalová A, Klánová J, Přibylová P, Váňa M (2015). Polycyclic aromatic hydrocarbons, polychlorinated biphenyls, and chlorinated pesticides in background air in central Europe - investigating parameters affecting wet scavenging of polycyclic aromatic hydrocarbons. Atmos Chem Phys.

[CR64] Shen L, Wania F (2005). Compilation, evaluation, and selection of physical-chemical property data for organochlorine pesticides. J Chem Eng Data.

[CR65] Škrdlíková L, Landlová L, Klánová J, Lammel G (2011). Wet deposition and scavenging efficiency of gaseous and particulate phase polycyclic aromatic compounds at a central European suburban site. Atmos Environ.

[CR66] Staelens J, de Schrijver A, van Avermaet P, Genouw G, Verhoest N (2005). A comparison of bulk and wet-only deposition at two adjacent sites in Melle (Belgium). Atmos Environ.

[CR67] Stemmler I, Lammel G (2012). Long-term trends of continental-scale PCB patterns studied using a global atmosphere-ocean general circulation model. Environ Sci Pollut Res.

[CR68] Teil MJ, Blanchard M, Chevreuil M (2004). Atmospheric deposition of organochlorines (PCBs and pesticides) in northern France. Chemosphere.

[CR69] UNECE (1999). Economic Commission for Europe. 1979 Convention on long-range transboundary air pollution and its 1998 protocols on persistent organic pollutants and heavy metals.

[CR70] UNEP (2003). United Nations Environment Programme. Regionally based assessment of persistent toxic substances, Global report 2003.

[CR71] UNEP (2008) United Nations Environment Programme. Stockholm Convention. http://chm.pops.int/Portals/0/download.aspx?d = UNEP-POPS-COP-CONVTEXT-2017.English.pdf (accessed April 4, 2019)

[CR72] Wania F, Westgate JN (2008). On the mechanism of mountain cold-trapping of organic chemicals. Environ Sci Technol.

[CR73] Wania F, Hoff JT, Jia CQ, Mackay D (1998). The effects of snow and ice on the environmental behaviour of hydrophobic organic chemicals. Environ Pollut.

[CR74] Wania F, Shen L, Lei YD, Teixeira C, Muir DCG (2003). Development and calibration of a resin-based passive sampling system for monitoring persistent organic pollutants in the atmosphere. Environ Sci Technol.

[CR75] Xiao H, Li NQ, Wania F (2004). Compilation, evaluation, and selection of physical-chemical property data for alpha-, beta-, and gamma-hexachlorocyclohexane. J Chem Eng Data.

