# Supplementary material for: Bulk atmospheric deposition of persistent organic pollutants and polycyclic aromatic hydrocarbons in Central Europe
Source: Environ Sci Pollut Res Int. 2019 Jun 14;26(23):23429–41. doi: 10.1007/s11356-019-05464-9 (PMC6667414; doi:10.1007/s11356-019-05464-9)
Supplement: Supplementary file 1 — (PDF 798 kb) [file 11356_2019_5464_MOESM1_ESM.pdf]

Supporting material

Bulk atmospheric deposition of persistent organic pollutants and polycyclic aromatic hydrocarbons in central Europe

Barbora Nežiková[1], Céline Degrendele[1], Pavel Čupr[1], Philipp Hohenblum[2], Wolfgang Moche[2], Roman Prokeš[1], Lenka Vaňková[1], Petr Kukučka[1], Jakub Martiník[1], Ondřej Audy[1], Petra Přibylová[1], Ivan Holoubek[1], Peter Weiss[2], Jana Klánová[1], Gerhard Lammel[1, 3] *

[1]Masaryk University, Research Centre for Toxic Compounds in the Environment, Brno, Czech Republic

[2]Umweltbundesamt, Wien, Austria

[3]Max Planck Institute for Chemistry, Multiphase Chemistry Department, Mainz, Germany

* Corresponding author. E-mail: g.lammel@mpic.de; Tel.: +49-6131-305-7600

Table of Content

S1. Methods

Table S1: Start and end of deposition sampling along with precipitation and temperature for the six sites.

Table S2: List of mean values and standard deviation of blanks for (a) PAHs, (b) PCBs and (c) OCPs.

Table S3: Instrumental limits of quantification (iLOQ) of (a) PAHs, (b) PCBs and (c) OCPs

Figure S1: Map of the sampling sites

Figure S2: The atmospheric deposition sampler used in this study presented as a scheme (a) and in field conditions (b)

S2. Results

Table S4: Bulk atmospheric deposition mass fluxes of SOC substance classes at (a) Košetice (KOS), (b) Kuchařovice (KUC), (c) Churáňov (CHU), (d) Wolkersdorf (WOL), (e) Unterbergern (UNT) and (f) Grünbach (GRU).

Figure S3: PAHs’, PCBs’ and OCPs’ particulate mass fraction in total deposition samples.

S1. Methods

Table S1: Start and end of deposition sampling along with precipitation and temperature for the six sites. Temperature are based on daily average. The meteorological data indicated for Unterbergern were obtained from a nearby meteorological station, Krems.

Location	Season	Sampling start	Sampling end	Precipitation (mm)	Temperature (K)
Kuchařovice (KUC) 48°52'52.6"N, 16°05'08.6"E	Autumn 2011	14/09/11	07/12/11	64.0	283.0
	Winter 2011-12	07/12/11	29/02/12	52.4	273.0
	Spring 2012	29/02/12	23/05/12	59.0	284.4
	Summer 2012	23/05/12	15/08/12	205.4	292.2
	Autumn 2012	15/08/12	07/11/12	125.6	286.4
	Winter 2012-13	07/11/12	13/02/13	87.0	273.5
	Spring 2013	13/02/13	08/05/13	134.3	276.7
	Summer 2013	08/05/13	31/07/13	209.0	288.8
Košetice (KOS) 49°34'24.73"N, 15°04'49.00"E	Autumn 2011	14/09/11	07/12/11	88.0	279.9
	Winter 2011-12	07/12/11	29/02/12	154.5	272.0
	Spring 2012	29/02/12	23/05/12	117.2	282.0
	Summer 2012	23/05/12	15/08/12	250.4	290.3
	Autumn 2012	15/08/12	07/11/12	159.8	283.5
	Winter 2012-13	07/11/12	13/02/13	174.1	272.8
	Spring 2013	13/02/13	08/05/13	130.4	277.5
	Summer 2013	08/05/13	31/07/13	296.8	288.5
	Autumn 2013	31/07/13	24/10/13	201.2	286.3
	Winter 2013-14	24/10/13	16/01/14	51.7	276.6
	Winter-Spring 2014	16/01/14	10/04/14	77.7	277.2
	Spring 2014	10/04/14	03/07/14	262.8	285.6
	Summer 2014	03/07/14	25/09/14	248.1	289.6
	Autumn 2014	25/09/14	18/12/14	84.4	280.2
	Winter 2014-15	18/12/14	12/03/15	91.2	273.9
	Spring 2015	12/03/15	04/06/15	92.6	282.2
	Summer 2015	04/06/15	27/08/15	168.0	292.0
Churáňov (CHU) 49°04'06.0"N, 13°37'02.0"E	Autumn 2011	14/09/11	07/12/11	143.5	280.2
	Winter 2011-12	07/12/11	29/02/12	300.9	273.4
	Spring 2012	29/02/12	23/05/12	138.2	283.1
	Summer 2012	23/05/12	15/08/12	447.8	292.1
	Autumn 2012	15/08/12	07/11/12	285.8	282.1
	Winter 2012-13	07/11/12	13/02/13	299.8	271.2
	Spring 2013	13/02/13	08/05/13	187.7	272.7
	Summer 2013	08/05/13	31/07/13	401.6	285.0
Wolkesdorf (WOL) 48°23'34.47"N, 16°31'18.98"E	Autumn 2011	14/09/11	07/12/11	69.8	281.3
	Winter 2011-12	7/12/2011	23/02/12	72.0	273.5
	Spring 2012	23/02/12	21/05/12	72.7	284.0
	Summer 2012	21/05/12	17/08/12	218.4	293.4

Unterbergern (UNT) 48°22'13.14"N, 15°32'43.76"E	Autumn 2011	16/09/11	05/12/11	58.5	280.2
	Winter 2011-12	05/12/11	29/02/12	44.6	273.4
	Spring 2012	29/02/12	24/05/12	101.1	283.1
	Summer 2012	24/05/12	20/08/12	216.7	292.1
Grünbach (GRU) 48°31'50.3"N, 14°34'30.2"E	Autumn 2011	14/09/11	06/12/11	121.8	279.9
	Winter 2011-12	06/12/11	27/02/12	26.8	269.5
	Spring 2012	27/02/12	22/05/12	62.7	280.0
	Summer 2012	22/05/12	15/08/12	277.7	288.6

Table S2: List of mean values and standard deviation of blanks for (a) PAHs, (b) PCBs and (c) OCPs. <iLOQ means that measured values were below instrumental limit of quantification.

(a)

Compound	Blank (ng sample^{-1})			
	GFF		XAD	
	Mean	Std. deviation	Mean	Std. deviation
ACY	<iLOQ		1.98	1.68
ACE	<iLOQ		1.65	1.48
FLN	<iLOQ		6.38	4.73
PHE	11.79	3.57	28.09	22.60
ANT	<iLOQ		<iLOQ	
FLT	7.70	0.46	13.42	11.72
PYR	2.58	0.23	4.78	4.10
BAA	0.40	0.07	0.53	0.35
CHR	1.32	0.21	1.85	1.47
BBF	<iLOQ		0.75	1.06
BKF	0.37	0.53	0.29	0.42
BAP	<iLOQ		<iLOQ	
IPY	<iLOQ		<iLOQ	
DHA	1.33	0.08	1.46	2.06
BPE	<iLOQ		<iLOQ	

(b)

Compound	Blank (pg sample^{-1})			
	GFF		XAD	
	Mean	Std. deviation	Mean	Std. deviation
PCB28	<iLOQ		<iLOQ	
PCB52	<iLOQ		<iLOQ	
PCB101	<iLOQ		<iLOQ	
PCB153	<iLOQ		<iLOQ	
PCB138	<iLOQ		<iLOQ	
PCB180	<iLOQ		<iLOQ	

(c)

**Compound**	**Blank (pg sample^{-1})**			
	GFF		XAD	
	Mean	Std. deviation	Mean	Std. deviation
PeCB	<iLOQ		<iLOQ	
HCB	<iLOQ		0.12	0.01
α-HCH	<iLOQ		<iLOQ	
β-HCH	<iLOQ		<iLOQ	
γ-HCH	<iLOQ		0.20	0.11
δ-HCH	<iLOQ		<iLOQ	
o,p'-DDE	<iLOQ		<iLOQ	
p,p'-DDE	<iLOQ		0.10	0.04
o,p'-DDD	<iLOQ		<iLOQ	
p,p'-DDD	<iLOQ		<iLOQ	
o,p'-DDT	<iLOQ		<iLOQ	
p,p'-DDT	<iLOQ		<iLOQ	

Table S3: Instrumental limits of quantification (iLOQ) of (a) PAHs, (b) PCBs and (c) OCPs

(a)

**Compound**	**iLOQ (ng sample^{-1})**	**iLOQ (ng sample^{-1})**
	For samples from 09/11 to 08/12	For samples from 08/12 to 08/15
ACY	0.50	0.50
ACE	0.50	0.79
FLN	0.50	0.38
PHE	0.50	0.16
ANT	0.50	0.35
FLT	0.50	0.35
PYR	0.50	0.27
BAA	0.50	0.35
CHR	0.50	0.28
BBF	0.50	0.72
BKF	0.50	0.79
BAP	0.50	0.85
IPY	0.50	0.68
DHA	0.50	0.79
BPE	0.50	0.45

(b)

Compound	iLOQ (pg sample^{-1})	iLOQ (pg sample^{-1})
	For samples from 09/11 to 08/12	For samples from 08/12 to 08/15
PCB28	0.05	0.07
PCB52	0.05	0.07
PCB101	0.05	0.05
PCB153	0.05	0.03
PCB138	0.05	0.06
PCB180	0.05	0.08

(c)

Compound	iLOQ (pg sample^{-1})	iLOQ (pg sample^{-1})
	For samples from 09/11 to 08/12	For samples from 08/12 to 08/15
PeCB	0.05	0.08
HCB	0.05	0.06
α-HCH	0.05	0.08
β-HCH	0.05	0.06
γ-HCH	0.05	0.08
δ-HCH	0.05	0.08
o,p'-DDE	0.05	0.06
p,p'-DDE	0.05	0.06
o,p'-DDD	0.05	0.07
p,p'-DDD	0.05	0.09
o,p'-DDT	0.05	0.07
p,p'-DDT	0.05	0.02

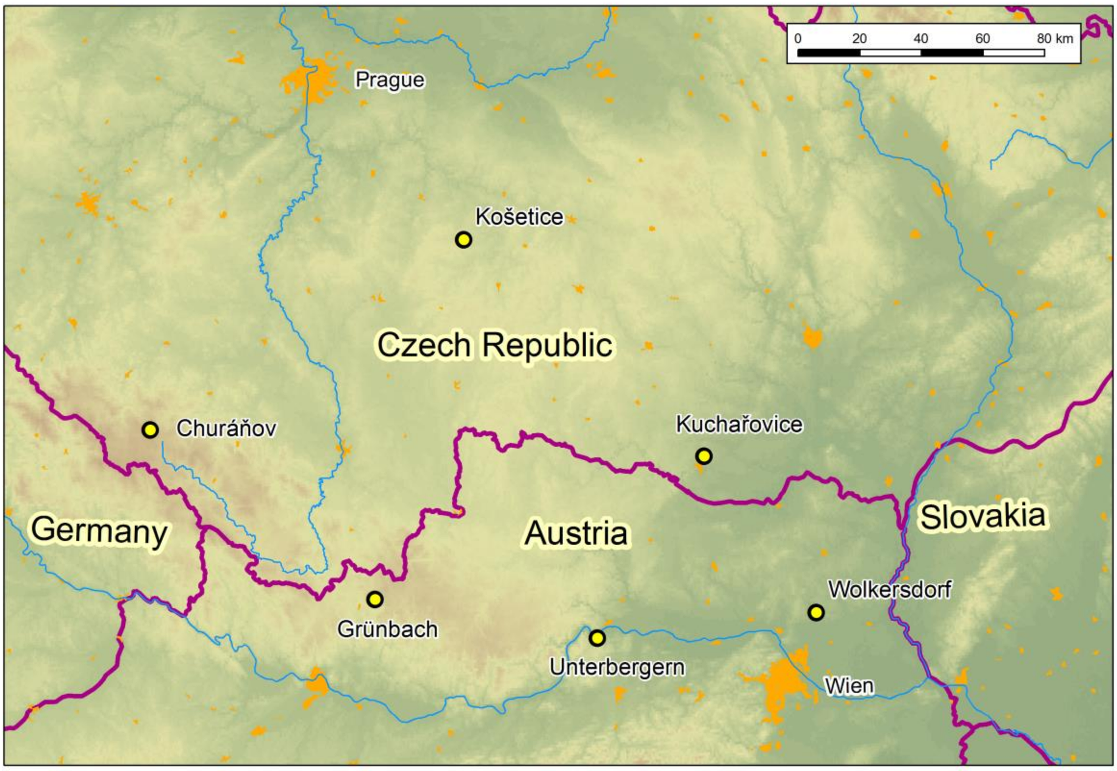

Figure S1: Map of the sampling sites

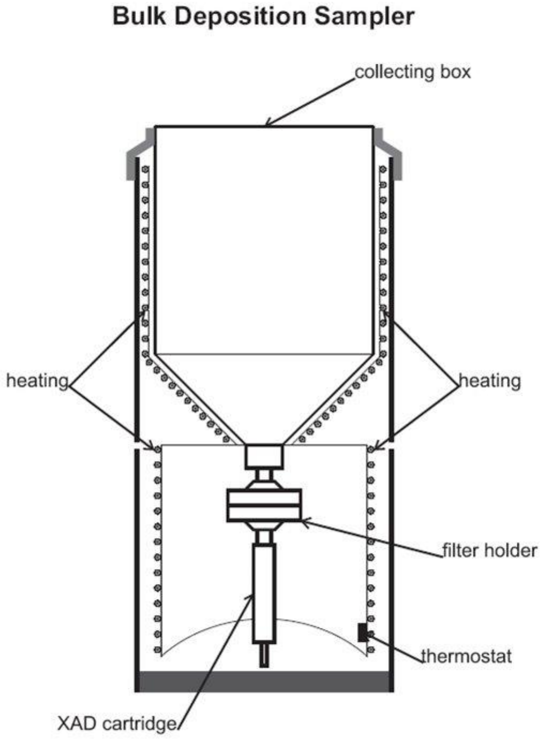

(a)

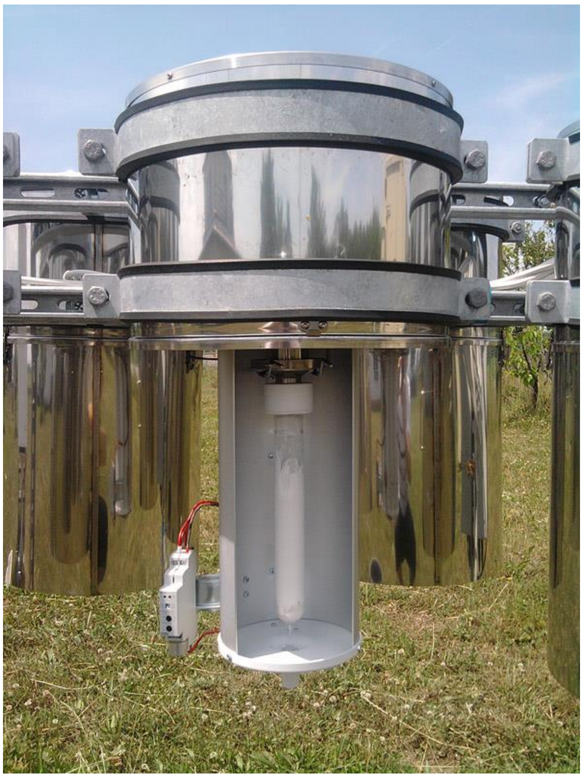

(b)

Figure S2: The atmospheric deposition sampler used in this study presented as a scheme (a) and in field conditions (b)

S2. Results

Table S4: Bulk atmospheric deposition mass fluxes of SOC substance classes at (a) Košetice (KOS), (b) Kuchařovice (KUC), (c) Churáňov (CHU), (d) Wolkersdorf (WOL), (e) Unterbergern (UNT) and (f) Grünbach (GRU).

(a)

	Σ_{15}PAHs (ng m^{-2} d^{-1})	Σ_6PCBs (pg m^{-2} d^{-1})	Σ_{12}OCPs (pg m^{-2} d^{-1})
Autumn 2011	41	160	1900
Winter 2011-12	140	440	2100
Spring 2012	29	130	1300
Summer 2012	40	2000	2600
Autumn 2012	84	99	420
Winter 2012-13	280	160	950
Spring 2013	210	110	1010
Summer 2013	120	270	2200
Autumn 2013	130	220	930
Winter 2013-14	57	170	670
Winter-Spring 2014	73	320	1700
Spring 2014	73	360	1500
Summer 2014	50	220	2600
Autumn 2014	83	240	1200
Winter 2014-15	120	260	1100
Spring 2015	120	460	610
Summer 2015	230	530	1400

(b)

	Σ_{15}PAHs (ng m^{-2} d^{-1})	Σ_6PCBs (pg m^{-2} d^{-1})	Σ_{12}OCPs (pg m^{-2} d^{-1})
Autumn 2011	49	64	2100
Winter 2011-12	190	160	1200
Spring 2012	1100	580	3100
Summer 2012	300	4400	7800
Autumn 2012	470	140	1700
Winter 2012-13	360	210	1100
Spring 2013	640	190	1700
Summer 2013	740	610	3700

(c)

	Σ_{15}PAHs (ng m^{-2} d^{-1})	Σ_6PCBs (pg m^{-2} d^{-1})	Σ_{12}OCPs (pg m^{-2} d^{-1})
Autumn 2011	25	230	1700
Winter 2011-12	49	160	970
Spring 2012	310	250	1500
Summer 2012	72	240	4600
Autumn 2012	100	94	2300
Winter 2012-13	160	200	1200
Spring 2013	410	140	1500
Summer 2013	35	120	1800

(d)

	Σ_{15}PAHs (ng m^{-2} d^{-1})	Σ_6PCBs (pg m^{-2} d^{-1})	Σ_{12}OCPs (pg m^{-2} d^{-1})
Autumn 2011	45	970	2100
Winter 2011-12	48	280	950
Spring 2012	48	720	410
Summer 2012	660	950	2500

(e)

	Σ_{15}PAHs (ng m^{-2} d^{-1})	Σ_6PCBs (pg m^{-2} d^{-1})	Σ_{12}OCPs (pg m^{-2} d^{-1})
Autumn 2011	31	350	2500
Winter 2011-12	23	290	1100
Spring 2012	180	330	1700
Summer 2012	75	78	1600

(f)

	Σ_{15}PAHs (ng m^{-2} d^{-1})	Σ_6PCBs (pg m^{-2} d^{-1})	Σ_{12}OCPs (pg m^{-2} d^{-1})
Autumn 2011	57	150	2300
Winter 2011-12	74	270	760
Spring 2012	220	250	920
Summer 2012	120	180	4100

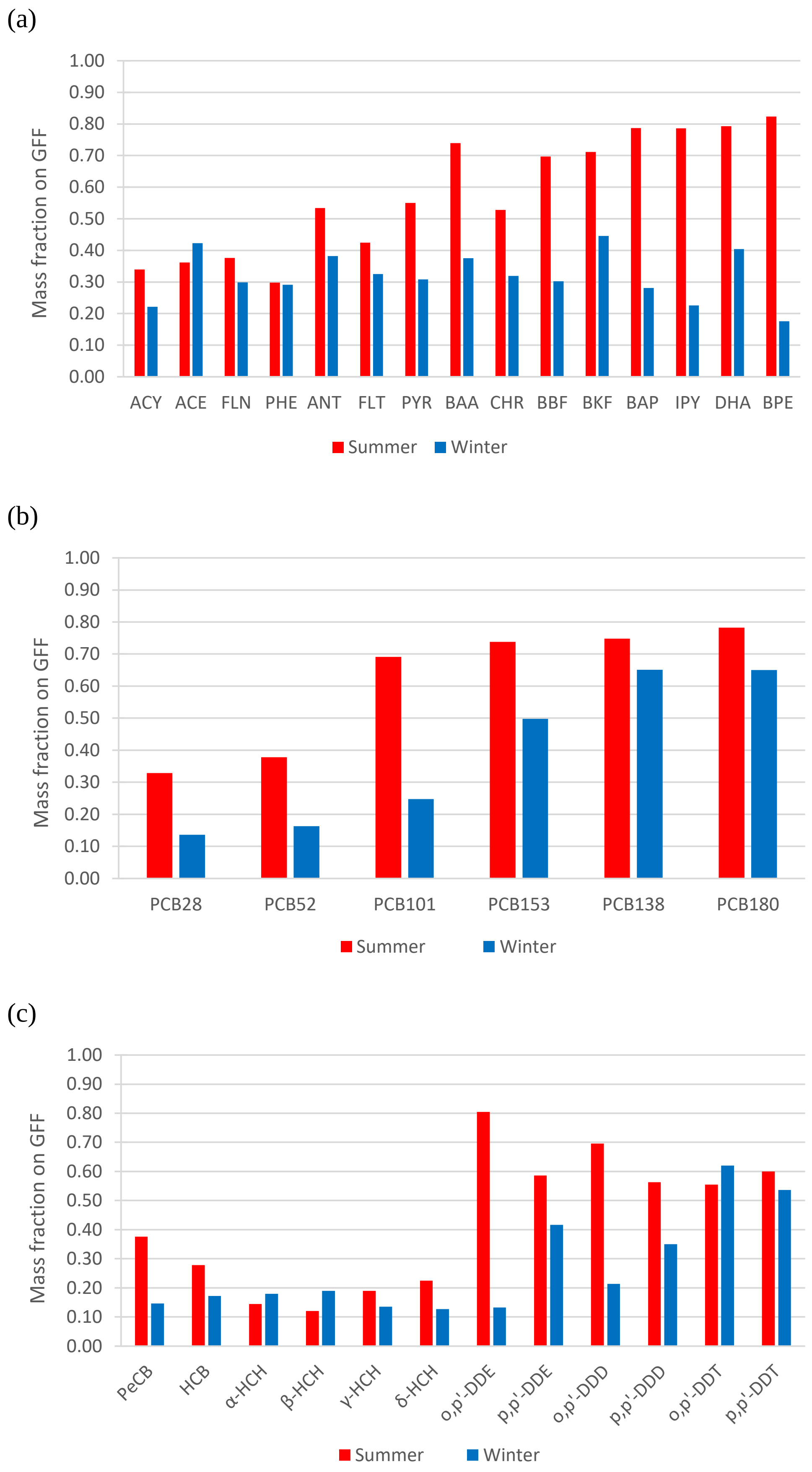

Figure S3: PAHs', PCBs' and OCPs' particulate mass fraction in total deposition samples.
